# Recurrent Hospitalisation in a Child With Hypopigmented Hair: Inborn Errors of Immunity Emerge

**DOI:** 10.7759/cureus.76554

**Published:** 2024-12-29

**Authors:** Rajkumar Kundavaram, Harish Kadiri, Ujjawal Khurana, Narendra K Chaudhary, Bhavna Dhingra, Umair Bargir, Shikha Malik

**Affiliations:** 1 Pediatrics, All India Institute of Medical Sciences, Bhopal, IND; 2 Pathology and Laboratory Medicine, All India Institute of Medical Sciences, Bhopal, IND; 3 Pediatric Immunology and Leukocyte Biology, National Institute of Immunohaematology, Mumbai, IND

**Keywords:** genetic analysis, griscelli syndrome, hypopigmented hair, inborn errors of immunity, pediatrics

## Abstract

Griscelli syndrome is a rare autosomal recessive disorder characterised by pigmentary dilution of skin and hair, recurrent skin and pulmonary infections, neurological manifestations, and immunodeficiency. We present a four-month-old female child with hypopigmented silvery hair and a history of recurrent hospitalisations for respiratory illness. The child was extensively evaluated for inborn errors of immunity (IEI), and the final diagnosis of type 2 Griscelli syndrome was made only after genetic testing. Antibiotic and antifungal prophylaxis was initiated, and the child is currently in good health. The family was counselled regarding haematopoietic stem cell transplant (HSCT) as the only curative option and the need for prenatal testing in further pregnancies. This case emphasises the need for a high index of clinical suspicion in diagnosing IEI and also highlights the limited therapeutic options and the role of genetic counselling.

## Introduction

Griscelli syndrome is a rare genetic disorder with autosomal recessive inheritance. This is characterised by partial albinism, neurological manifestations, and immunologic defects. According to the Genetic and Rare Disease Information Centre, only 160 cases have been reported in the literature as of December 2024. Griscelli et al. first reported about this syndrome in 1978 [1]. The disease is heterogeneous, with three different phenotypes due to three different genes on chromosome 15q21. The products of these three genes facilitate the transport of melanocytes. Mutations in these genes result in characteristic hypopigmentation, a common feature among all three types.

Type 1 Griscelli syndrome is due to *MYO5A* gene mutations and is characterised by severe neurological features with characteristic skin and hair colouring [2]. Developmental delay, intellectual disability, seizures, and hypotonia are the clinical manifestations. Elejalde disease closely mimics type 1 Griscelli syndrome, with many of the same signs and symptoms previously regarded as a separate entity now included as a part of type 1 Griscelli syndrome. Type 2 Griscelli syndrome is the most common of all three forms due to mutations in *RAB27A* and is characterised by neurologic involvement, autoinflammatory phenomenon, and dermatological manifestations [3]. These patients can also have haemophagocytic lymphohistiocytosis (HLH) as a complication, making the course challenging. Type 3 Griscelli is due to melanophilin (*MLPH*) gene mutations with dermatological manifestations alone and no neurological or immune phenomenon with a very good prognosis [2]. We present the case of a child with hypopigmentation and immunodeficiency diagnosed with type 2 Griscelli syndrome by genetic analysis. This case was presented as a poster at the 6th National Conference on Inborn Errors of Immunity, Chandigarh, India, on November 27, 2022.

## Case presentation

**Figure 1:**
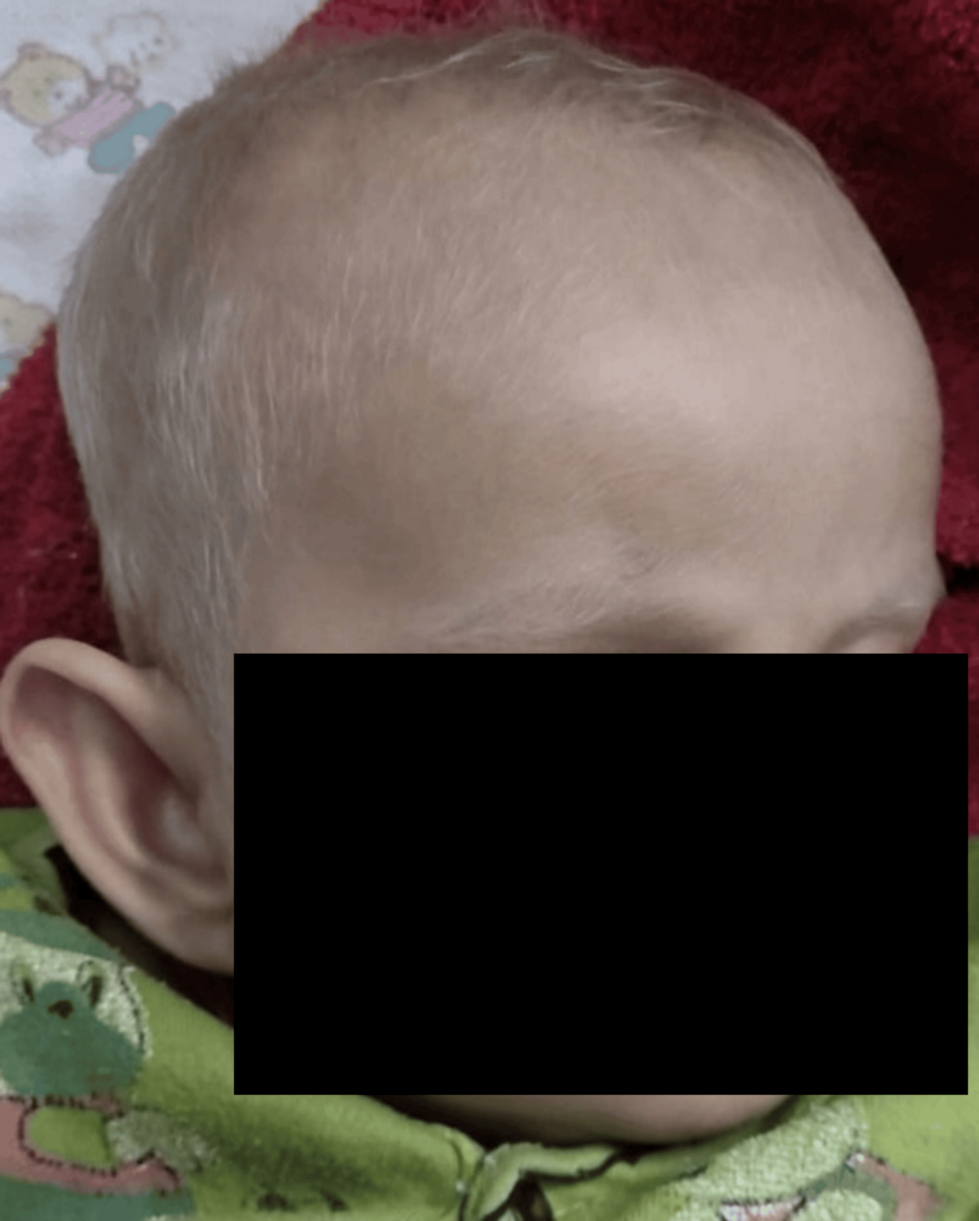
Silvery scalp hair and eyebrows

A four-month-old female child presented with a history of recurrent hospitalisations for febrile episodes requiring the administration of intravenous antibiotics. The child was born of a non-consanguineous marriage and was symptomatic by 15 days of life, requiring four hospital admissions for respiratory illness. The elder sibling died at two years of age with a history of seizures, hypopigmented hair, and behavioural problems. Magnetic resonance imaging (MRI) revealed leukodystrophy, which was evaluated at another institute. On physical examination, the child had pallor with no obvious facial dysmorphism. The child had silvery hair on the scalp and eyebrows (Figure 1).

Her growth indices were normal. Systemic examination revealed hepatosplenomegaly, whereas the brain examination was unremarkable. In view of recurrent infections requiring hospital admissions and administration of IV antibiotics and significant positive history, a possibility of inborn errors of immunity (IEI) was considered. Our differential diagnoses were Chediak-Higashi syndrome, Elejalde syndrome, Griscelli syndrome, and Hermansky-Pudlak syndrome.

Initial laboratory parameters revealed anaemia (haemoglobin: 8.8 g/dL, mean corpuscular volume: 69 fL) with raised leucocyte counts, neutropenia and lymphocytosis for age (total leucocyte count (TLC): 16,940/mm^3^, absolute neutrophil count (ANC): 1610/mm^3^, absolute lymphocyte count (ALC): 14,150/mm^3^). A peripheral smear did not reveal any specific neutrophilic granules. In view of low ANC for age, a serum immunoglobulin assay was conducted (Table 1).

**Table 1:** Haemogram and immunoglobulin profile of the patient TLC: total leucocyte count; ANC: absolute neutrophil count; ALC: absolute lymphocyte count

Parameter	Value	Reference Range
Haemoglobin (g%)	8.8	8–17
TLC (×10^3^/mm^3^)	16.9	3–15
ANC (×10^3^/mm^3^)	1.61	1.5–7.0
ALC (×10^3^/mm^3^)	14.1	1–3.7
Platelets (lakh/mm^3^)	1.76	1.5–4.0
Immunoglobulin A (mg/dL)	110	100–400
Immunoglobulin G (mg/dL)	1279	700–1600
Immunoglobulin M (mg/dL)	265	50–200

Further evaluation of IEI was planned, and lymphocyte subset analysis was performed, which did not show a deficiency of any lymphocyte subset (Table 2).

**Table 2:** Lymphocyte subset analysis NK cells: natural killer cells

Lymphocytes	Patient Value % (Absolute Count/mm^3^)	Reference Range % (Absolute Count/mm^3^)
T cell (CD3+)	62.44	66.3 ± 7.6 (700-2100)
CD4 T cell (CD3+/CD4+)	15.6	34.4 ± 6.7 (300-1400)
CD8 T cell (CD3+/CD8+)	43.63	27.8 ± 6.3 (200-900)
B cell (CD 19+)	15.25	14.1 ± 4.9 (100-500)
NK cell (CD16+/CD56+)	21.11	15.4 ± 6.3 (90-600)

**Figure 2:**
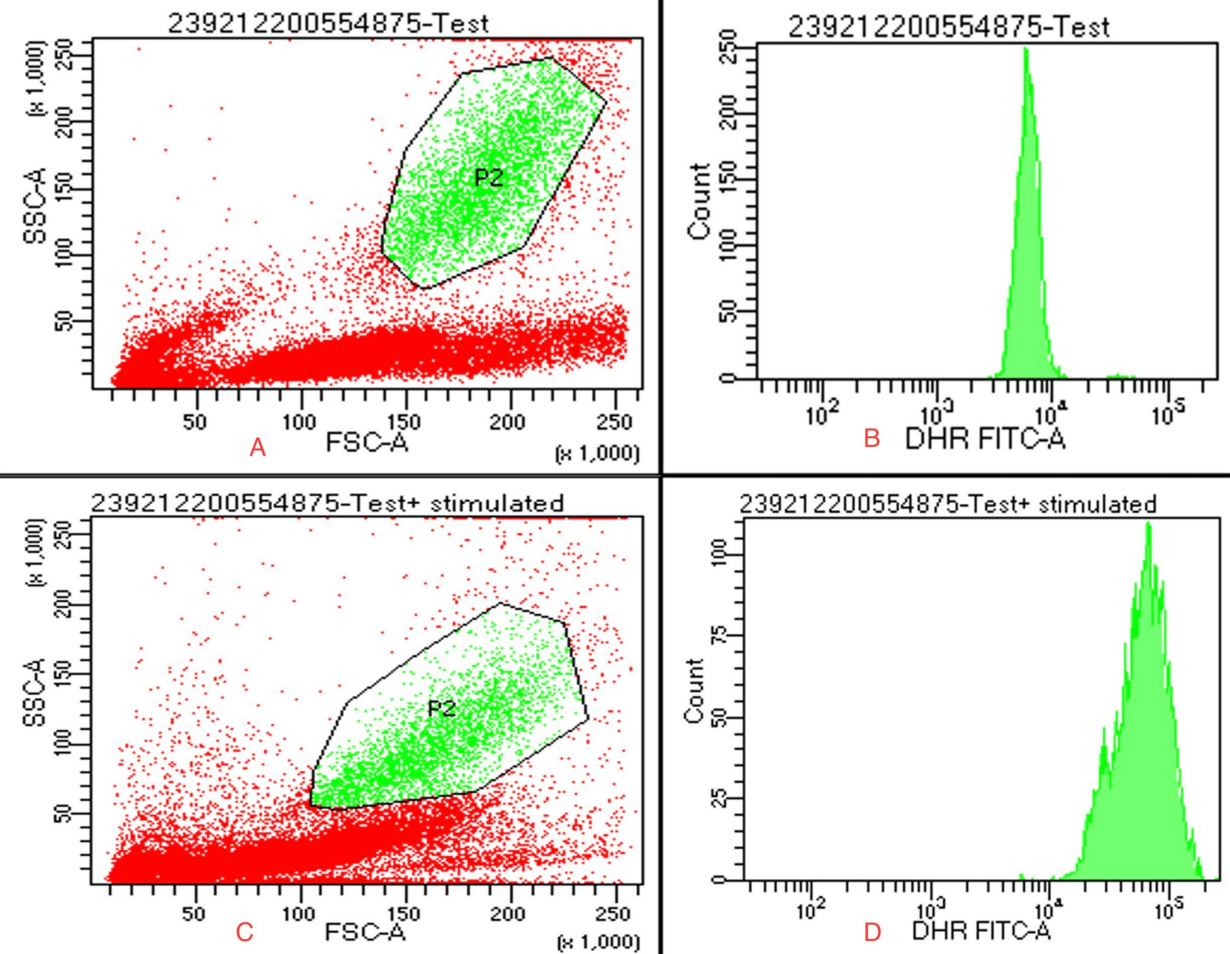
Dihydrorhodamine test showing a normal oxidative burst of neutrophils (A) Forward scatter versus side scatter P2 represents neutrophils, unstimulated; (B) cytometric histogram showing rhodamine fluorescence of unstimulated neutrophils; (C) forward scatter versus side scatter P2 represents neutrophils, stimulated; (D) cytometric histogram showing rhodamine fluorescence of stimulated neutrophils. FSC: forward scatter; SSC: side scatter; DHR: dihydrorhodamine; FITC: fluorescein isothiocyanate

To rule out chronic granulomatous disease, a dihydrorhodamine test was done, and the results were normal (Figure 2).

**Figure 3:**
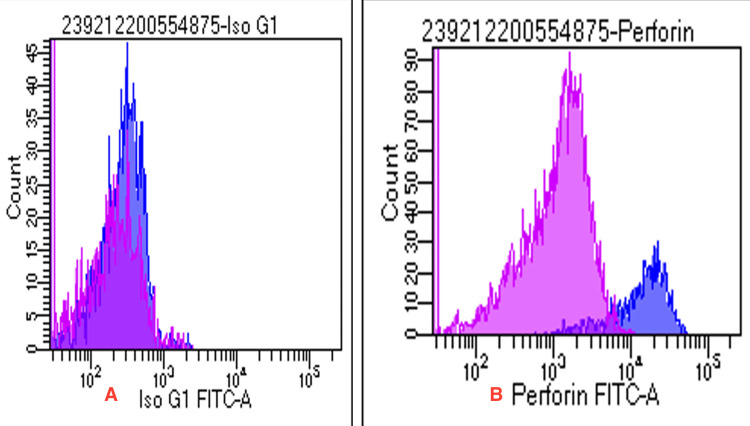
Flow cytometry showing a normal perforin expression (A) Control; (B) test (purple represents CD8 T cells, whereas violet denotes NK cells). FITC: fluorescein isothiocyanate

Flow cytometry confirmed normal perforin expression (Figure 3).

On microscopic examination of the hair, large clumps of pigment were seen all along the shaft. Bone marrow aspiration and biopsy were normal; histological findings from the skin punch biopsy of the scalp were consistent with oculocutaneous albinism. Genetic analysis revealed a defect in *the RAB27A* gene, confirming the diagnosis of Griscelli syndrome type 2 (Table 3).

**Table 3:** Genetic analysis confirming RAB27A mutation OMIM: Online Mendelian Inheritance in Man

Gene & Transcript	Variant	Location	Zygosity	Disorder (OMIM)	Inheritance	Classification
RAB27A NM_004580.5	c.258_260dupAGC (p.Ala87dup)	Exon 4	Homozygous	Griscelli syndrome (type 2) (607624)	Autosomal recessive	Likely pathogenic
G6PD NM_001042351.3	c.949G>A (p.Glu317Lys)	Exon 9	Heterozygous	G6PD deficiency (300908)	X-linked recessive	Pathogenic
CYP21A2 NM_000500.9	c.293-13C>G	Intron 2	Heterozygous	Adrenal hyperplasia, congenital, due to 21-hydroxylase deficiency (201910)	Autosomal recessive	Pathogenic

The child responded well to intravenous antibiotics and was discharged with antifungal prophylaxis. The child is doing well on the follow-up visit after six months. Parents are counselled for prenatal diagnosis in the next pregnancy and haematopoietic stem cell transplant (HSCT) as the only curative treatment.

## Discussion

All three types of Griscelli syndrome have pigment dilution: silvery-grey hair, eyebrows, and eyelashes. The distribution of hair pigment and skin biopsy findings will help differentiate Griscelli syndrome from other silvery hair syndromes but will not differentiate Griscelli syndrome subtypes. Genetic analysis is the definitive diagnosis of a specific type.

Mutations in the *RAB27A* gene lead to Griscelli syndrome type 2 [4]. This gene encodes for guanosine triphosphate (GTP)-binding GTPase, which is involved in transporting melanosomes [5]. Defective *RAB27A* leads to abnormal melanosome distribution. Another function of *RAB27A* is to promote the secretion of exosomes, and mutations lead to defective exocytosis of cytotoxic granules of NK cells and cytotoxic T lymphocytes. Thus, Griscelli syndrome type 2 is characterised by specific dermatological manifestations, variable neurological involvement, and immunodeficiency. The clinical course is complicated by immunological phenomena and HLH. The only available curative option is HSCT. A timely diagnosis can alter clinical outcomes, as preparing the child and family for HSCT before the onset of HLH will improve survival.

Skin manifestations

Dermatological involvement is characterised by silvery-grey hair, eyebrows, and eyelashes. The skin also develops a characteristic bronze pigmentation after exposure to the sun. Small and large irregular pigment clumps are seen on light microscopy examination of the hair shaft. A monotonous whitish appearance is seen on polarised microscopy. Histopathology of the skin shows a basal epidermis layer composed of irregularly distributed pigment with dense granules with no melanosomes in keratinocytes. Cutaneous granulomas can also be the presentation, possibly due to HLH [6]. In our case, the child presented with characteristic silvery hair, eyebrows, and eyelashes.

Immunodeficiency

Type 2 Griscelli syndrome has combined immunodeficiency (both humoral and cell-mediated). These patients have low serum immunoglobulins, low isohaemagglutinins, and an impaired vaccine response. The mechanism of immunodeficiency is due to defective exosome secretion [5]. There is also impaired T cell function with complete skin anergy, and the proliferative response to mitogens is normal. Patients also have reduced cytotoxic activity even though normal content of perforins and granzymes is seen. The mechanism of granule release is defective, and hence, these patients are prone to HLH. Our patient was brought to medical attention because of impaired immunity, making her susceptible to repeated respiratory infections. These patients are also prone to developing immune complications after immunisation with live vaccines and can predispose to HLH. In our patient, we have advised the parents not to get the child immunised with live vaccines.

Neurological manifestations

The most common neurologic manifestation is seizure and can be focal or generalised, including status epilepticus [7]. Auditory, visual, and language deficits can also be seen to varying degrees. Cerebellar signs are also frequently reported [8]. Signs and symptoms of raised intracranial tension (ICT), intracranial haemorrhage, encephalopathy, motor deficit, or hemiparesis can also be presenting manifestations. As there is no expression of *RAB27A* in neural cells, the neurologic impairment is not due to mutations in *RAB27A* but secondary to central nervous system (CNS) lymphohistiocytic infiltration and granuloma formation, i.e. neurologic involvement can occur despite systemic HLH [9].

Meeths et al. described in 2009 that 67% of patients with *RAB27A* mutations exhibited neurologic manifestations during the disease, with more than half presenting at the time of diagnosis [10]. The MRI of the brain reveals hyperintense lesions in T2-weighted or fluid attenuation inversion recovery (FLAIR) sequences, close differential to demyelinating diseases, and patients might be initially diagnosed with acute disseminated encephalomyelitis or multiple sclerosis [11]. Cerebrospinal fluid (CSF) analysis can be normal or may show pleocytosis or elevated protein levels. Histopathology of neurologic lesions shows lymphohistiocytic infiltration and/or granulomas. CNS involvement is associated with increased mortality, and survivors suffer from neurologic sequelae. However, in our patient, no specific neurological manifestations are observed.

In patients exhibiting silvery hair syndromes accompanied by neurological symptoms, a clinical approach alone is not sufficient to distinguish between the two types in the absence of immunodeficiency or HLH. The differentiation between the two types is important since HSCT can improve neurologic alterations in type 2 but not in type 1. The definitive approach to making a diagnosis is by genetic analysis. However, the easy availability of genetic studies is a concern, and the sequencing of *RAB27A* alone may not detect all the pathogenic variants as *RAB27A* only has five small coding exons with relatively short introns [12]. Despite most of the pathogenic variants being described within these five exons, there are some reports of duplications and deletions in patients with Griscelli syndrome.

Since early diagnosis is critical for treatment and outcome, other approaches, such as functional assays in cytotoxic lymphocytes or RAB27A protein expression levels, can be undertaken to establish a molecular diagnosis [13]. All patients suspected of Griscelli syndrome should undergo functional tests for NK cell degranulation to determine impaired cytotoxicity compatible with Griscelli syndrome type 2, and preparations for pre-emptive HSCT should be initiated.

## Conclusions

Griscelli syndrome, although rare, should be suspected in children presenting with albinism and immunodeficiency. It is not possible to differentiate three subtypes on clinical grounds, and genetic analysis is the only method to differentiate. Type 2 is complicated by immunodeficiency and HLH, and the long-term prognosis is poor. The only definitive curative option is HSCT, but success rates are variable. If this is done before the onset of HLH, survival rates can be improved. Genetic counselling is a crucial part of management, and prenatal diagnosis in subsequent pregnancies should be emphasised. Parents should also be informed regarding the need for antibiotic prophylaxis and warned against immunisation with live vaccines.
